# Two-year effectiveness of erenumab in resistant chronic migraine: a prospective real-world analysis

**DOI:** 10.1186/s10194-022-01507-8

**Published:** 2022-11-04

**Authors:** Anna P. Andreou, Matteo Fuccaro, Bethany Hill, Madeleine Murphy, Valeria Caponnetto, Rachael Kilner, Giorgio Lambru

**Affiliations:** 1 Institute of Psychiatry, Psychology and Neuroscience, Headache Research-Wolfson CARD, King’s College London, London, UK; 2 Headache and Facial Pain Service, Guy’s and St Thomas’ NHS Foundation Trust, London, UK; 3 Department of Applied Clinical Sciences and Biotechnology, University of L’Aquila, L’Aquila, Italy

**Keywords:** Erenumab, Chronic migraine, Resistant migraine, CGRP, Monoclonal antibodies

## Abstract

**Background:**

Controlled and real-world evidence have demonstrated the efficacy of calcitonin gene related peptide (CGRP) monoclonal antibodies (MABs) in migraine. However, data on the over-one-year sustained effectiveness of CGRP MABs in resistant chronic migraine (CM) is sparse.

**Methods:**

This is a two-year real-world prospective analysis of an ongoing single centre audit conducted in patients with resistant CM. Patients received monthly erenumab for six months before assessing its effectiveness. Responders were considered those who achieved at least 30% reduction in monthly migraine days (MMD) by month 6, compared to baseline. Secondary outcomes were also analysed, including changes of the Headache Impact Test version 6 (HIT-6).

**Results:**

One hundred sixty-four patients [135 (82.3%) females; mean age 46 SD 14) years] were included in the audit and 160 patients analysed. Patients had failed a mean of 8.4 preventive treatments at baseline. At month 6, 76 patients (48%) were 30% responders to erenumab, 50 patients (31%) were 50% responders and 25 (15%) were 75% responders. The mean reduction in MMD at month 6 was 7.5 days compared to baseline (*P* < 0.001). At month 12 and month 18, 61 patients (38%) and 52 patients (33%) remained 30% responders respectively. At month 24, 36 patients (23%) remained 30% responders, 25 patients (16%) and 13 patients (8%) were respectively 50% and 75% responders. Compared to 95% of patients at baseline, at months 6, 12 and 24, 46%, 29% and 16% of responders respectively had severe disability. At least one adverse event at month 6, 12, 18 and 24 was reported by 49%, 19%, 11% and 3% of patients. By month 6, 13% of patients discontinued the treatment because of side effects, often constipation.

**Conclusions:**

Long-term sustained effectiveness of erenumab was reported only by a minority of resistant CM patients. Although more research in resistant migraine is needed, Erenumab can provide long-term meaningful reduction in migraine load and migraine-related disability in some patients.

## Introduction

The term difficult-to-treat migraine (also called “resistant” or “refractory”) is an evolving definition still under debate. The term refers to the subgroup of episodic or chronic migraine patients whose symptoms do not respond to established preventive treatments. These patients experience poor quality of life, they often struggle to keep full-time employments and need frequent access to healthcare systems for symptoms management [1, 2]. The main variable used to define this patients’ subgroup is the number of treatments failed with thresholds that vary between 2–4 classes of treatments [1]. A recent expert consensus on this topic suggested the use of the term “resistant” for patients who failed at least three classes of migraine preventatives and suffer from at least eight debilitating headache days per month for at least three consecutive months without improvement and the term “refractory” those who failed all of the available preventatives and suffer from at least eight debilitating headache days per month for at least six consecutive months [3]. The introduction of the monoclonal antibodies (MABs) targeting the calcitonin gene-related peptide (CGRP) or its receptor has expanded the arsenal of migraine preventive treatments, giving new hopes to the “refractory” migraine population. These treatments have shown superiority to placebo in reducing mean monthly migraine days (MMD) and improving in migraine-related quality of life scales in clinical trials across the different migraine subtypes [4–7]. Real-world studies have confirmed the short-term clinical effectiveness of this new class of medication in both migraine and difficult-to-treat migraine [7–10]. Promising data has demonstrated the long-term safety and efficacy of erenumab (Aimovig™), a fully human monoclonal antibody, and the only one against the CGRP receptor, for the prevention of episodic migraine (EM) [11–13]. However sparse data is available on the long-term efficacy and safety of the CGRP MABs in the difficult-to-treat CM population. Given the complexity of the management of migraine symptoms in this population, gathering long-term data may help clarifying the long-term clinical relevance of inhibiting the CGRP pathway in this subgroup of patients and the extent of the sustained effect of this class of drug in managing symptoms long-term and improving migraine-related disability in refractory CM [14].

Erenumab was made available free-of charge in the United Kingdom (UK) for the prevention of CM in patients who failed at least three preventive treatments, as part of an agreement between Novartis and the National Health System (NHS) Trusts across the UK. Subsequently, in March 2021, the National Institute for Health and Care Excellence (NICE) UK approved the use of erenumab in adults with at least four migraine days per month who failed at least three preventive treatments [15]. We have previously reported the 6-month effectiveness and tolerability of erenumab in large real-world difficult-to-treat CM population [9]. Here, we present the two-year follow-up extension analysis of this ongoing clinical audit.

## Methods

This is the continuation of a registered prospective clinical audit, part of the CGRP service evaluation, conducted at the Headache Service at Guy’s and St Thomas’ NHS Foundation Trust, London, UK. Audit under current national guidelines does not require research ethics committee review [16].

**Fig. 1:**
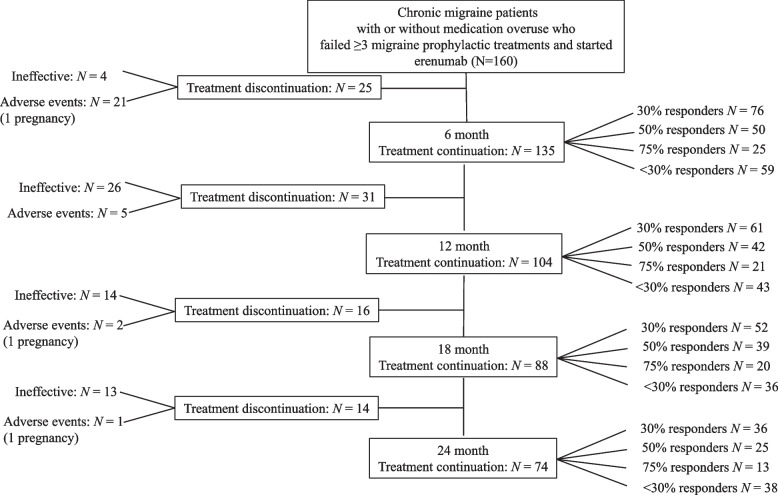
Audit design and patients treatment pathway

This analysis aims to evaluate the long-term effectiveness and tolerability of erenumab in adults with difficult-to-treat CM. The audit design is presented in Fig. 1. In brief, adult patients meeting the International Headache Society (IHS) criteria for CM who failed at least three preventive treatments were included in the audit. The most commonly used medications included: propranolol, amitriptyline, topiramate, candesartan, pizotifen, gabapentin, pregabalin, flunarizine, greater occipital nerve blocks, single-pulse transcranial magnetic stimulation (sTMS) and onabotulinum toxin A (BoNT/A). Patients with medication overuse headache (MOH) were not excluded from the analysis. Patients were prescribed monthly subcutaneous injections of erenumab using the pre-filled autoinjector for a total of six months before establishing efficacy. All patients received at least three 70 mg erenumab injections performed one month apart. By month 6 all patients had increased the dose to 140 mg. Patients were asked to complete a daily headache diary, monthly Headache Impact Test-6 (HIT-6) questionnaire and to report any side effects that occurred during the treatment period. Patients were followed-up in the headache nurses-led CGRP clinic at month 3, month 6, followed-up by the consultant at month 12 and subsequently in the CGRP clinic at month 18 and 24. The cut-off for treatment continuation was set at least 30% reduction of migraine days after 12 weeks of treatment [15].

In line with the NICE UK recommendation [15], the main efficacy outcomes were changes from baseline in the mean MMD at months 6, 12, 18 and 24. Secondary efficacy outcomes included: changes from baseline in mean monthly headache days (MHD), change in mean monthly headache-free days and changes in mean monthly abortive treatment intake days. Changes in the proportion of patients achieving at least a 30%, 50% and 75% reduction in their mean MMD was also evaluated (respectively 30%, 50%, 75% responders). To assess whether any change in efficacy measures was associated with improvement in migraine-related disability, change in HIT-6 score were analysed. A “headache day” was defined as a day with headache lasting for ≥ 4 h and with a severity of ≥ 4/10 on a verbal rating scale (0 no head pain, 10 worst pain ever experienced). A “migraine day” was defined according to the IHS classification criteria [17]. A “headache-free day” was defined as a day without any head pain. An “abortive treatment intake day” was considered any day where patients consumed abortive treatments for attempted headache relief. The cut-off outcome treatment continuation past the 6-month time point was reduction in the mean MMD of at least 30%, in line with the NICE guidelines and recommendations from the chronic pain clinical trials consensus [15, 18]. The number of patients reporting adverse events (AEs) at each time point was calculated. For patients who discontinued the treatment, reason for discontinuation and subsequent treatment plan was included.

### Statistical analysis

All outcomes pre- and post-erenumab treatment were measured on a continuous scale. For all measures considered here, data demonstrated a skewed distribution with a significant deviation from normal distribution (Kolmogorov–Smirnov test; *P* < 0.05). As a result, the Wilcoxon signed ranks test was used to compare the change in values over time. For independent group comparison the Mann–Whitney test was used. *P*-values of less than 0.05 were regarded as evidence of a statistically significant result. Effects of erenumab between and within groups were analysed using SPSS (Statistical Package for Social Science) version 23 (IBM, USA). Any missing values were treated in SPSS as discrete missing values. All data are provided as mean ± standard deviation (SD), unless stated otherwise. Where relevant, patient numbers have additionally been given as a percentage of all registered patients.

## Results

### Demographic and baseline headache characteristics

A total of 164 patients initiated the erenumab subcutaneous treatment during the audit period [135 (82.3%) females; mean age 46 SD 14) years]. At the time of analysis for this report, accurately filled monthly diaries and HIT-6 at baseline and at the set follow-ups were available for 160 patients, which were included in the analysis. All patients were medically resistant according to the European Headache Federation (EHF) consensus [3], with the average number of failed preventive treatments being 8.3 ± 3.6 and average duration of CM of 12.1 ± 11 years. The majority of patients failed to respond to BoNT/A injections (91.9%), while all patients failed to obtain a meaningful response to greater occipital nerve blocks (GONBs). A percentage of 54.4% of patients had MOH at baseline. Demographic and clinical characteristics of the patients’ group at baseline are summarised in Table 1.

**Table 1:** Demographic and clinical characteristics at baseline of all chronic migraine patients treated with erenumab (*N* = 160)

**Variables**	
Females, n (%)	132 (82.5%)
Age (y), mean ± SD	48 ± 14.3
CM duration (y), mean ± SD	12.1 ± 11.0
Aura, n (%)	53 (33.1%)
Medication overuse, n (%)	87 (54.4%)
Monthly migraine days, mean ± SD	19.8 ± 9.0
Monthly headache days, mean ± SD	23.4 ± 7.3
Monthly headache free days, mean ± SD	3.8 ± 5.7
Monthly abortive treatment intake days, mean ± SD	11.6 ± 8.8
HIT-6 score, mean ± SD	67.6 ± 4.9
Number of preventive treatments failed, mean ± SD	8.3 ± 3.6
BoNT/A non-responders, n (%)	147 (91.9%)

*BoNT/A* onabotulinum toxinA, *CM* Chronic migraine, *HIT-6* Headache Impact Test version 6, *n* number, *SD* Standard deviation, *y* years

### Short- and long-term efficacy outcomes

Overall, the mean reduction in MMD at month 3 was 6.0 days (*P* = 0.002). At that time point, 49%, 35% and 13% of patients obtained at least a 30%, 50% and 75% reduction in MMD, respectively. Overall, of the 160 patients who received erenumab, 135 patients (84%) continued to receive the treatment until month 6. At month 6, the mean reduction in monthly migraine days for all patients was 7.5 days at month 6 (*P* < 0.001) compared to baseline, and 48% of patients (*n* = 76) achieved a reduction of at least 30% of their MMD and continued the treatment. Of them, 50 patients (31.3%) achieved at least a 50% reduction in MMD and 25 (15.6%) achieved at least a 75% reduction in MMD. No patient became completely migraine/headache-free during the treatment. Compared to baseline, the mean reduction of HIT-6 score was 7.7 points at month 3 (from 67.6 ± 0.4 to 59.9 ± 0.9; *P* < 0.001) and 7.5 points at month 6 (60.1 ± 1.3; *P* = 0.01). The percentage of patients with MOH was reduced from 54% at baseline to 20% at month 3 and to 25% at month 6.

At month 12, 61 patients (38%) patients maintained at least a 30% reduction in MMD, 42 patients (26%) achieved at least a 50% reduction in MMD, while 21 of them (13%) achieved at least a 75% reduction in MMD. At month 18, 52 patients (33%) maintained at least a 30% reduction in MMD. Similar to outcomes of month 12, 39 patients (24%) maintained at least a 50% reduction in their MMD and 20 patients (13%) maintained at least a 75% reduction in their MMD.

**Fig. 2:**
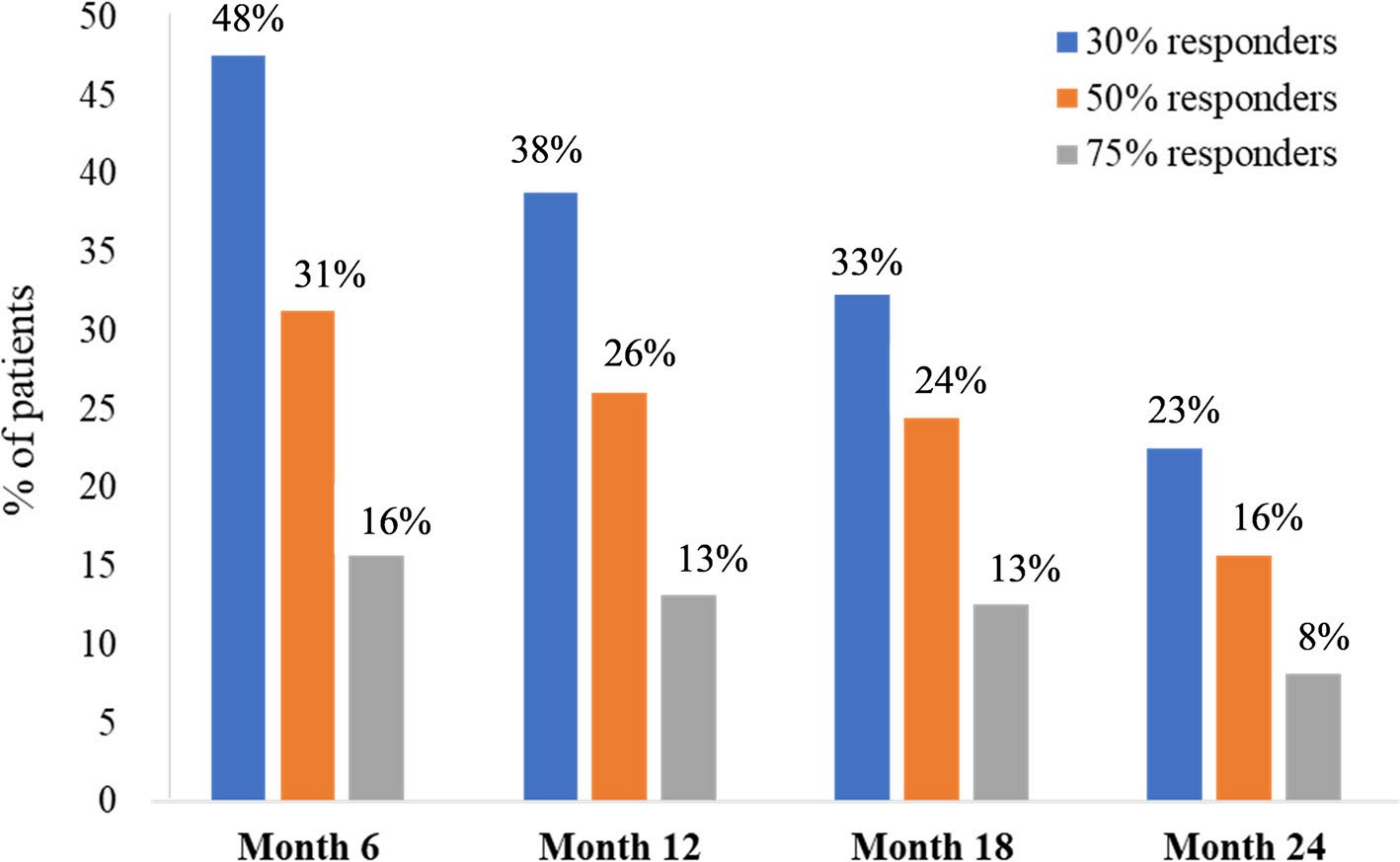
Percentages of 30, 50, and 75% responders to erenumab at different time points

At month 24, 36 patients (23%) patients maintained at least a 30% reduction in MMD, 25 patients (16%) reported at least a 50% reduction in MMD, while 13 patients (8%) maintained at least a 75% reduction in their MMD. Figure 2 outlines the percentage of patients who achieved at least 30%-50%-75% mean reduction in MMD (30%-50%-75% responders) at months 6, 12, 18 and 24 post treatment initiation.

Amongst the 30% responders, the reduction in mean MMD between baseline and month 24 was 10 days (from 19.8 ± 9.0 to 8.8 ± 8.0). The other migraine clinical and disability outcomes were also significantly improved compared to baseline. The mean changes in efficacy and disability outcomes are outlined in Table 2.

**Table 2:** Clinical characteristics at baseline for all patients and at 6, 12, 18 and 24 months for 30% responders to erenumab

	**Baseline**	**Month 6**	**Month 12**	**Month 18**	**Month 24**
	**(*n* = 160)**	**(*n* = 76)**	**(*n* = 61)**	**(*n* = 52)**	**(*n* = 36)**
MD	19.8 ± 9.0	6.9 ± 5.2^*^	9.1 ± 7.7^*^	8.7 ± 8.7^*^	8.8 ± 8.0^*^
HD	23.4 ± 7.3	11.0 ± 8.2^*^	12.0 ± 8.5^*^	12.0 ± 9.6^*^	11.3 ± 8.7^*^
Headache free days	3.8 ± 5.7	7.8 ± 8.2^**^	11.5 ± 10.1^**^	11.6 ± 10.3^**^	13.3 ± 10.3^**^
Abortive treatment intake days	11.6 ± 8.8	5.6 ± 5.9^*^	7.4 ± 7.8^**^	6.4 ± 6.8^**^	6.2 ± 5.1^**^
HIT-6	67.6 ± 4.9	56.7 ± 7.7^**^	57.5 ± 9.0^**^	58.0 ± 9.6^**^	54.5 ± 8.2^**^

All values are expressed in mean monthly numbers

*HD* Headache days, *MD* Migraine days, *MMD* Monthly migraine days

^*^P < 0.001, ^**^P < 0.05

At baseline, 95% of patients fell within the severe HIT-6 impact category. Of the patients who continued the treatment, at months six, twelve and twenty-four 46%, 29% and 16% of patients fell within the severe HIT-6 impact category.

Due to the lack of other evidence-based treatment options at the time of the audit, some patients who did not reach 30% reduction in MMD (*n* = 59 patients) were offered treatment continuation. The majority of these patients had reported a fluctuating 20–29% reduction in MMD by month 6. At months 12, 18 and 24, the number of patients who continued the treatment with less than 30% reduction in MMD was 43, 36 and 38, respectively.

### Treatment discontinuation

Between months one and 24, out of the 160 patients who were included, 86 (54%) discontinued the treatment. By month six, four patients discontinued the treatment because of inefficacy and 21 due to side effects (including one pregnancy). At the month six assessment, another 28 patients stopped the treatment due to lack of efficacy (*n* = 23) or due to persistent adverse events (*n* = 5), while by month 12 three more patients stopped the treatment due to lack of consistent efficacy. Between months 13 and 18, 16 further patients discontinued the treatment;14 because of inefficacy and two because of side effects, including one pregnancy. By month 24, further 14 patients discontinued the treatment, 13 patients because of inefficacy and one because of pregnancy.

### Safety and tolerability

In the first six months of the treatment, nearly 50% patients reported at least one adverse event (79/160). Of the patients who reported side effects, the most frequent adverse events were constipation in 34 patients (21.2%) and flu-like symptoms in 25 patients (15.6%). Although adverse events were transient, lasting up to two weeks post-injection and described as mild or moderate in the great majority of patients, 21 patients discontinued erenumab due to adverse events in the first 6 months; among these, one patient discontinued due to pregnancy. Severe adverse events leading to treatment discontinuation in the first six months were constipation (*n* = 11), headache worsening after each injection (*n* = 5), flu-like symptoms (*n* = 2), whole body itchiness (*n* = 1), severe mood deterioration (*n* = 1) and new onset hypertension (*n* = 1). Between months 6 and month 12, further five patients discontinued the treatment due to persistent side effects. These included constipation (*n* = 4), generalised body aches and fatigue (*n* = 1). After month 12, only one patient stopped the treatment due to treatment related constipation. At months 12, 18 and 24 the number of patients reporting at least one side effect diminished as follows: 30 (28.8%), 18 (20.5%) and 4 (5.4%) respectively, with constipation remaining the most frequently reported side effect. Overall, amongst all patients there were 167 side effects reported up to month 24. A full list of adverse events reported at any time point can be found in Table 3.

**Table 3:** Percentage and types of adverse events in patients treated with erenumab at month 6, 12, 18 and 24

	**Month 6**	**Month 12**	**Month 18**	**Month 24**
At least one adverse event, N (%)	79 (49.4%)	30 (18.8%)	18 (11.3%)	4 (2.5%)
**Type of events (number)**				
Constipation	34	21	13	4
Flu-like symptoms	25	1	0	0
Body aches	10	2	1	0
Itchiness	8	1	0	0
Fatigue	6	5	1	0
Injection site reaction	5	0	2	0
Muscle spasms	3	1	0	0
Nausea	2	1	0	0
Excessive sweating	2	0	0	0
Joint stiffness or pain	1	3	1	0
Weight gain	1	1	1	0
Dry mouth	1	1	0	0
Dizziness	1	1	0	0
Headache worsening	1	0	1	0
Arterial hypertension	1	0	0	0
Mental health deterioration	1	0	0	0
Tremor	1	0	0	0
Night terrors	1	0	0	0
Flashes	0	1	0	0
Total	104	39	20	4

Three female patients became pregnant during the erenumab treatment (one during the first four months of treatment, one after 13 months of treatment and one after 17 months of treatment), and hence the treatment was discontinued. Pregnancies were reported without complications and no health issues were reported in new-borns and in postpartum mothers.

## Discussion

This prospective analysis explored the 24-month effectiveness and tolerability of an anti-CGRP MAB in the difficult-to-treat CM population. The findings of our analysis showed that almost half of the patients exposed to erenumab for six months were 30% responders, about 1/3 of patients were 50% responders and about 1/7 of patients were 75% responders. The 30% responders to erenumab at month 24 maintained a meaningful improvement in migraine symptoms and migraine-related disability as shown by the reduction in the mean HIT-6 score. However, less than 1/4 of the initial patients population were 30% responders at 24 months follow-up. This figure equates to about half of the 30% responders at month 6. This 50% drop in responders was also observed overtime in the 50% and 75% responders categories. These findings were less impressive than in recently published studies testing erenumab in difficult-to-treat migraine. In a 2-year open label analysis of erenumab in the treatment of difficult-to-treat episodic migraine, the 50% response rate was maintained by a significant percentage of the initial study group [13]. This positive trend was largely maintained in the 5-year follow-up analysis of the same population of patients [19]. A 1-year follow-up open-label subgroup analysis of difficult-to-treat CM suggested a long-term sustained beneficial effect of erenumab regardless of the number of preventive classes failed by the patients [12]. A recently published real-world large study testing erenumab in 300 difficult-to-treat CM patients, showed an initially promising effect of erenumab with 71% of patients experiencing at least a 30% reduction in MMD. However, overtime its beneficial effect declined and at 1-year follow-up, only 34% remained 30% responders [8]. This finding is more in line with our long-term experience with erenumab in resistant CM patients, which showed a progressive decline in its beneficial effect so that only about 1/3 of the overall population was still responders after 1 year of treatment and less than 1/ 4 of patients were responders after 2 years. It is arguable that differences in studies methodologies between open-label continuation of RCTs and real-world studies may explain the different discontinuation rate due to treatment inefficacy. Differences between clinical trial and real-life populations in terms of migraine subtypes may also play a role [20]. Most clinical trials testing anti-CGRP MABs in the treatment of difficult-to-treat CM included patients who failed of 2–4 classes of preventive treatments. However, most patients failed 2–3 classes of treatment and only a small percentage of patients failed four preventive treatments at baseline [6, 7]. Conversely, real-world patients have often failed several treatments and have complex medical histories and comorbidities. Our patients failed an average of eight preventive treatments, greater occipital nerve blocks and almost all patients failed at least two onabotulinum toxin A treatments.

It is difficult to explain how the inhibition of such a pivotal migraine pathway produces short-term meaningful benefits which overtime become less apparent in the majority of patients treated, at least in the resistant CM population. It is arguable that other mechanisms may be unmasked by the inhibition of CGRP and may become more relevant in sustaining the migraine load in the complex population of resistant CM. More research needs to clarify whether the number of failed treatments, genetic mechanisms, patients’ comorbidities or other variables play a role in determining refractoriness in migraine and other headache disorders [21].

It has been shown that erenumab treatment may lead to a slowly progressive improvement overtime in mean MMD, suggesting a cumulative effect of the medication in some responders and perhaps a disease-modifying effect [11]. Our data did not show a clear cumulative effect overtime in responders in terms of changes in mean MMD. The greatest degree of mean MMD reduction in our patients occurred by month 6 though. A real-world study on erenumab in difficult-to-treat migraine suggested that a percentage as high as 13.5% with no response to three months of treatment with erenumab, obtained a 50% response between the fourth to the sixth dose [10]. Similar percentages were shown in our previous 6 month real-world analysis [9]. Taken together, this data may suggest that at least in the resistant CM population, six months of treatment with erenumab (and generally with CGRP pathway inhibitors) rather than three, may be more appropriate, given the complexity of these patients and their refractoriness to treatments. It is also noteworthy that continuing treatment in patients who have not reached the 30% reduction in mean MMD after six months of erenumab, is unlikely to produce a delayed response respond.

Patients who do not respond or stop responding to an anti-CGRP MAB are considered “refractory” as the EHF definition. It remains to be demonstrated whether this group of patients will show a sustained respond to a different anti-CGRP MAB. Moreover, it could be postulated that the proportion of refractory CM long-term non-responders to erenumab may benefit from polytherapy. Preliminary data suggest that the combination of anti-CGRP MABs and Onabotulinum toxin A may be clinically superior to each treatment in monotherapy [22]. Hopefully more data on combination therapy between CGRP modulators and other treatments will emerge in the future and maybe able to offer more treatment options to the refractory CM patients.

Similarly to other real world studies, the frequency of adverse events in our study is higher than in clinical trials [5, 8–10, 23]. However, the discontinuation rate due to side effects in our patients was 13.1% at month 6, which is higher than both clinical trials and some of the real-world studies [10, 11, 23, 24]. Patients’ selection differences has been postulated as one of the main explanations [8]. Constipation emerged as a very frequent side effect, which can sometimes lead to treatment discontinuation. It is advisable to council patients about to start erenumab particularly about this side effect. Interestingly, the longer patients were exposed to erenumab, the fewer side effects were reported. This has important implication in clinical practice when counselling patients before starting erenumab or during treatment continuation.

Pre-clinical studies suggested the involvement of CGRP in the regulation of utero-placental blood flow and uterine relaxation. The anti-CGRP MABs can cross the placenta after the first 20–22 weeks of pregnancy. Sparse data has been published on the effect of anti-CGRP MABs on pregnancy. Thus far, no specific maternal toxicities, patterns of major birth defects, or increased reporting of spontaneous abortion were reported. No safety issues also emerged in our three patients, though caution needs to be kept until much larger database data will be published [25].

Our analysis has some limitations, including the audit design. However the audit was conducted as rigorously as possible, and in a systematic manner by collecting objective clinical information from migraine diaries and HIT-6 scores. Also the audit allowed us to include a real-world group of difficult-to-treat migraine, which reflects headache practice in tertiary headache clinics. Another limitation is the open label design. However, the progressive reduction in responders, likely indicated the lack of a long-term placebo effect. The main strengths of this analysis include the evaluation of the effectiveness of erenumab up to two years follow-up in a complex migraine population with a great unmet need for more research on better treatments.

## Conclusion

Despite promising short-term clinical effectiveness in resistant CM population, the 2-year sustained effectiveness of erenumab was observed in less than 1/ 4 of patients. This finding suggests that the majority of resistant CM may not benefit from long-term treatment with erenumab, highlighting the need for better understanding of the long-term relevance of CGRP pathway inhibition in CM migraine treatment. More targeted research is needed to shed light on the neurobiological mechanisms involved in migraine refractoriness. Nonetheless Erenumab can be effective in some resistant CM patients, in whom it provides long-term meaningful reduction in migraine load and migraine-related disability.

## Data Availability

Anonymized data are available from the corresponding author upon reasonable request.

## Abbreviations

AEs: Adverse events
BoNT/A: Onabotulinum toxin A
CGRP: Calcitonin gene related peptide
CM: Chronic migraine
EHF: European Headache Federation
GONBs: Greater occipital nerve blocks
HIT-6: Headache Impact Test version 6
IHS: International Headache Society
MABs: Monoclonal antibodies
MHD: Monthly headache days
MMD: Monthly migraine days
MOH: Medication overuse headache
NHS: National Health System
NICE: National Institute for Health and Care Excellence
RCTs: Randomised-control trials
SD: Standard deviation
SPSS: Statistical Package for Social Science
UK: United Kingdom
